# Improved operative and recovery times with mini-thoracotomy aortic valve replacement

**DOI:** 10.1186/s13019-019-0912-0

**Published:** 2019-05-09

**Authors:** Anna Olds, Siavash Saadat, Anthony Azzolini, Viktor Dombrovskiy, Karen Odroniec, Anthony Lemaire, Aziz Ghaly, Leonard Y. Lee

**Affiliations:** 1 Division of Cardiothoracic Surgery, Rutgers Robert Wood Johnson Medical School, New Brunswick, NJ USA; 2 Boston, USA

**Keywords:** Valvular heart disease, Aortic valve disease, Valve surgery, Minimally invasive surgery

## Abstract

**Background:**

The small incisions of minimally invasive surgery have the proposed benefit of less surgical trauma, less pain, and faster recovery. This study was done to compare minimally invasive techniques for aortic valve replacement, including right anterior mini-thoracotomy and mini-sternotomy, to conventional sternotomy.

**Methods:**

We retrospectively reviewed 503 patients who underwent isolated aortic valve replacement at our institution from 2012 to 2015 using one of three techniques: 1) Mini-thoracotomy, 2) Mini-sternotomy, 3) Conventional sternotomy. Demographics, operative morbidity, mortality, and postoperative complications were compared.

**Results:**

Of the 503 cases, 267 (53.1%) were mini-thoracotomy, 120 (23.8%) were mini-sternotomy, and 116 (23.1%) were conventional sternotomy. Mini-thoracotomy patients, compared to mini-sternotomy and conventional sternotomy, had significantly shorter bypass times [82 (IQ 67–113) minutes; vs. 117 (93.5–139.5); vs. 102.5 (85.5–132.5), respectively (*p* < 0.0001)], a lower incidence of prolonged ventilator support [3.75% vs. 9.17 and 12.9%, respectively (*p* = 0.0034)], and required significantly shorter ICU and postoperative stays, resulting in an overall shorter hospitalization [6 (IQ 5–9) days; vs. 7 (5–14.5); vs 9 (6–15.5), respectively (*p* < 0.05)]. Incidence of other postoperative complications were lower in the mini-thoracotomy group compared to mini-sternotomy and conventional sternotomy, without significance. Minimally invasive techniques trended towards better survival [mini-thoracotomy 1.5%, mini-sternotomy 1.67%, and conventional sternotomy 5.17% (*p* = 0.13)].

**Conclusions:**

Minimally invasive aortic valve replacement approaches are safe, effective alternatives to conventional sternotomy. The mini-thoracotomy approach showed decreased operative times, decreased lengths of stay, decreased incidence of prolonged ventilator time, and a trend towards lower mortality when compared to mini-sternotomy and conventional sternotomy.

## Background

Conventional full sternotomy had been the standard approach to aortic valve replacement for many years, with a known perioperative risk of 1–4% in all age groups throughout the world [1]. Since the late 1990s, minimally invasive techniques for aortic valve surgery, including mini-sternotomy and anterior mini-thoracotomy, have been gaining acceptance [2].

Claims of reduced postoperative complications, length of stay, and mortality have been based on studies comparing conventional aortic valve surgery to minimally invasive techniques [3–8]. The small incisions of minimally invasive surgery have the proposed benefit of less surgical trauma, leading to less postoperative bleeding, fewer blood transfusions, and a decreased incidence of deep sternal wound infections [1, 3, 9, 10]. Further, patients generally experience less pain and more rapid postoperative recovery, along with an improved cosmetic outcome, all of which promote faster rehabilitation and expedited return to normal activities [5]. Finally, forgoing the use of conventional sternotomy theoretically allows for safer reentry in cases of reoperation. Therefore, many believe that minimally invasive aortic valve replacements have evolved into an efficient treatment option in experienced centers, providing greater patient satisfaction and lower complication rates [7, 11]. Some studies, however, have shown that smaller incisions lead to poor exposure, potentially making the surgery more difficult and more dangerous, with longer operative times [12]. In this study we compare outcomes and perioperative variables after minimally invasive aortic valve replacements, via a mini sternotomy or an anterior thoracotomy approach, in comparison to outcomes after conventional sternotomy.

## Methods

### Patient selection

This study was approved by the Rutgers Robert Wood Johnson Medical School Institutional Review Board, meeting all ethical and legal requirements without the need for the acquisition of informed consent. All preoperative data, in-hospital outcomes and post-discharge outcomes were collected from the medical records and the Robert Wood Johnson University Hospital Cardiac Surgery Database according to The Society of Thoracic Surgeons (STS) National Adult Cardiac Database version 2.81 definitions.

This study is a retrospective review involving 503 patients ≥18 years of age who underwent isolated surgical aortic valve replacement (AVR) at Robert Wood Johnson University Hospital between January 2012 and December 2015 using one of three techniques: 1) Mini-thoracotomy (MT), 2) Mini-sternotomy (MS), 3) Conventional sternotomy (CS). We excluded patients who had other simultaneous operations, and any pregnant patients.

For most surgeons in the practice, all patients are considered candidates for mini-thoracotomy isolated AVR via right anterior mini-thoracotomy. If there is a contraindication to mini-thoracotomy due to anatomical reasons or body habitus (mini-thoracotomy AVR may be difficult in morbidly obese patients), mini-sternotomy or full sternotomy is the second choice within our practice. The mini-sternotomy approach was performed in the early years of our experience with minimally invasive AVR. Subsequently, most isolated AVRs are done using mini-thoracotomy because it provides a faster recovery, improved cosmetic outcome, and patients generally prefer the mini-thoracotomy approach.

### Data collection

Demographics, pre-operative comorbidities, STS scores, and aortic valve dysfunction were compared, along with operative morbidity and mortality, length of hospital stays, and postoperative complications. Patients were followed for 30 days after hospital discharge. Length of stay is reported as an aggregate number that includes preoperative and postoperative lengths of stay. Postoperative complications included prolonged ventilator time (defined as total number of ventilator hours > 24 h), postoperative atrial fibrillation, postoperative renal failure, reoperation for bleeding, deep sternal infection, and postoperative stroke.

### Surgical procedure

At our center, mini-thoracotomy AVR has become the standard of care for most patients undergoing isolated AVR. Recently, we have begun expanding the patient population that qualifies for mini-thoracotomy AVR, and we have begun including higher risk patients. The right anterior mini-thoracotomy approach was completed through a 6 cm incision in the 2nd intercostal space, while the upper mini-sternotomy incision was carried down to the 3rd or 4th intercostal space. Full median sternotomy was performed according to standard technique. Cardiopulmonary bypass (CPB) was established by femoral arterial or direct ascending aortic cannulation, and percutaneous femoral venous or direct right atrial cannulation. Aortic cross clamp was applied through the incision or through a separate stab wound to accommodate the clamp. Anterograde cardioplegia was administered in standard fashion and/or directly into the coronary ostia after aortotomy. Retrograde cardioplegia was utilized through a coronary sinus cannula placed via the right atrial appendage, or via the right internal jugular vein, using transesophageal echocardiography (TEE), and a left ventricular vent was placed through the aortic valve or the right superior pulmonary vein. The aortic valve replacement was performed in a standard fashion. The choice of valve was at the discretion of the surgeon. For all full sternotomy, mini-sternotomy, and mini-thoracotomy patients, the CorKnot (LSI Solutions, Victor, NY) is used to tie down the valve and facilitates the ease of valve placement somewhat in the setting of limited exposure in mini-thoracotomy cases. No automated sutures or other similar technologies were used. Air was evacuated from the heart under trans-esophageal echocardiographic (TEE) guidance. After surgery, all patients were transferred to the cardiothoracic surgical intensive care unit and managed according to unit protocol. All surgeries were performed by the same group of 4 surgeons. The mini-thoracotomy approach is not performed by all surgeons, in which case the mini-sternotomy would be performed.

### Post-operative care

Our institution’s protocol for full sternotomy cases involves extubating patients in the intensive care unit (ICU) postoperatively. For minimally invasive cases, our protocol has evolved over the past few years. Currently, the protocol involves extubating patients in the operating room at the termination of surgery. Prior protocols involved later extubation. Following extubation, these patients are then taken to the cardiac ICU. Chest tubes and Foley catheters are removed in the morning on postoperative day 1.

We have recently implemented fast track protocols for ICU and hospital discharge since our center has increased the number of minimally invasive cases. These protocols constitute aggressive early transfer out of the ICU and an early discharge policy for all minimally invasive cases, with some patients discharged to home in as little as two or three days. In most instances, minimally invasive cases stay in the ICU for 6 h and are then transferred to the step-down floor. Most elective patients are now discharged home on postoperative day 2 or 3.

### Statistical analysis

SAS 9.4 software (SAS Institute, Cary, NC) was used for data analysis and statistics. Intergroup differences were performed with the Chi-square or Fisher’s exact test for categorical variables. Continuous variables were tested for normality of distribution with the use of the Kolmogorov-Smirnov test. Results for non-normally distributed ones were presented as median with interquartile range (IQR: 25th–75th percentiles) and were compared by nonparametric Wilcoxon rank sum test. Multivariable logistic regression analysis was performed for main outcomes, including mortality, prolonged ventilator time, and postoperative complications, with adjustment for demographic characteristics and preoperative conditions. Two-tailed *P* < 0.05 was considered significant.

## Results

Patient demographics and type of aortic valve pathology are presented in Table 1. Of the 503 cases during the study period, 267 (53.1%) were mini-thoracotomy, 120 (23.8%) mini-sternotomy, and 116 (23.1%) conventional sternotomy (Table 1). There were 16 patients converted to conventional sternotomy (CS) to facilitate increased visibility and access, including 10 (3.75%) in the mini-thoracotomy (MT) group, and 6 (5.0%) in the mini-sternotomy (MS) group (Table 2); the difference was not statistically significant (*p* = 0.57).

**Table 1:** Preoperative characteristics

		Surgical Approach		
	MT (*n* = 267)	MS (*n* = 120)	CS (*n* = 116)	*P*-value
Patients, n (%)	267 (53)	120 (23.9)	116 (23)	
Age (y), median (IQR)	75 (67–81)	73.5 (66–80.5)	74 (62.5–80)	0.48
Gender				0.98
Male, n (%)	153 (57.3)	69 (57.5)	68 (58.6)	
Female, n (%)	114 (42.7)	51 (42.5)	48 (41.4)	
STS Score (%), median (IQR)	2.1 (1.1–4)	2.7 (1.6–4.7)	3.0 (1.9–6.0)	< 0.01
Current smoker, n (%)	14 (5.2)	8 (6.7)	12 (10.3)	0.02
Prior smoker, n (%)	128 (47.9)	66 (55)	56 (48.3)	0.26
Diabetic, n (%)	77 (28.8)	44 (36.7)	43 (37.1)	0.16
Hypertension, n (%)	240 (89.9)	105 (87.5)	95 (81.9)	0.1
Hyperlipidemia, n (%)	214 (80.1)	101 (84.2)	94 (81.0)	0.64
Chronic Renal Failure, n (%)	9 (3.4)	8 (6.7)	5 (4.3)	0.35
Cerebrovascular Disease, n (%)	38 (14.2)	21 (17.5)	16 (13.8)	0.66
Past Cerebrovascular Accident, n (%)	19 (7.12)	10 (8.3)	11 (9.5)	0.72
Congestive Heart Failure, n (%)	98 (36.7)	41 (34.2)	42 (36.2)	0.89
Ejection Fraction (%), median (IQR)	58 (53–60)	56 (48–63)	57 (49.5–63.3)	0.47
Aortic Stenosis, n (%)	246 (92.1)	109 (90.8)	101 (87.1)	0.14
Aortic Insufficiency				0.18
None, n (%)	110 (41.2)	44 (36.7)	46 (39.7)	
Trace, n (%)	17 (6.37)	13 (10.8)	9 (7.8)	
Mild, n (%)	50 (18.7)	34 (28.3)	31 (26.7)	
Moderate, n (%)	57 (21.3)	21 (17.5)	17 (14.7)	
Severe, n (%)	33 (12.4)	8 (6.7)	13 (11.2)	

Abbreviations: *MT* Mini-thoracotomy, *MS* Mini-sternotomy, *CS* conventional sternotomy, *IQR* interquartile range, *STS Score* Society of Thoracic Surgeons Score

**Table 2:** Perioperative Parameters and Postoperative Outcomes

		Surgical Approach		
	MT (n = 267)	MS (n = 120)	CS (n = 116)	p-value
Valve Type				0.21
Bioprosthetic Valve, n (%)	261 (97.8)	118 (98.3)	110 (94.8)	
Mechanical Valve, n (%)	6 (2.25)	2 (1.67)	6 (5.17)	
CPB time (min), median (IQR)	82 (67–113)	117 (94–140)	103 (86–133)	0.0001
Aortic X-clamp (min), median (IQR)	58 (48–85)	91 (69–108)	71 (57–100)	0.0001
Conversions, n (%)	10 (3.8)	6 (5.0)	N/A	0.59
ICU LOS (hours), median (IQR)	22 (17–31)	25 (18–49)	31 (22–68)	< 0.05
Postop LOS (days), median (IQR)	5 (4–7)	6 (4–9)	6 (4–10)	< 0.05
Total LOS (days), median (IQR)	6 (5–9)	7 (5–15)	9 (6–16)	< 0.05
Prolonged Vent time, n (%)	10 (3.8)	11 (9.2)	15 (12.9)	< 0.01
Stroke, n (%)	2 (0.8)	1 (0.8)	1 (0.9)	1.0
Reoperation, n (%)	7 (2.6)	6 (5.0)	3 (2.6)	0.44
Atrial fibrillation, n (%)	62 (23.2)	37 (30.8)	32 (27.6)	0.26
Renal Failure, n (%)	8 (3.0)	6 (5.0)	6 (5.2)	0.44
Deep Sternal Infection, n (%)	0	0	0	
30-day Mortality, n (%)	4 (1.5)	2 (1.7)	6 (5.2)	0.13

Abbreviations: *MT* Mini-thoracotomy, *MS* Mini-sternotomy, *CS* conventional sternotomy, *CPB* cardiopulmonary bypass, *IQR* interquartile range, *ICU* intensive care unit, *LOS* length of stay, *Postop* postoperative

Preoperative STS scores were significantly different among the three groups (*p* = 0.0084). Patients who underwent mini-thoracotomy had significantly lower STS scores than patients who underwent conventional sternotomy (*p* = 0.0028). There was no difference in STS scores between MT and MS groups (*p* = 0.0956), or between MS and CS groups (*p* = 0.2495).

Both aortic cross clamp time and cardiopulmonary bypass time were significantly shorter in the MT group than in the other two groups (Table 2). Median aortic cross clamp time was significantly shorter in the MT group than in the MS (*p* < 0.0001) and CS (*p* = 0.0001) groups. Median cardiopulmonary bypass time was significantly shorter in the MT group when compared to both the MS (p < 0.0001) and CS (*p* < 0.0001) groups. Alternatively, aortic cross clamp and bypass times were significantly longer in the MS group than in the CS group (*p* = 0.0003 and *p* = 0.0242, respectively).

All lengths of stay reported as ICU length of stay, postoperative length of stay, and total hospital length of stay, were shorter in the MT group (Table 2). The MT group required a shorter ICU stay when compared to the MS (*p* = 0.0033) and CS (p < 0.0001) groups. The MT group also demonstrated shorter postoperative length of stay than the MS (*p* = 0.0857) and CS (*p* = 0.0002) groups, and shorter median total LOS than the MS (*p* = 0.0093) and CS (p < 0.0001) groups. The MS group had significantly shorter total LOS when compared to the CS group (*p* = 0.0197).

All postoperative complications were less common in the MT group as compared to the other two groups (Table 2). Analysis of postoperative complications revealed that the MT approach had a significantly lower incidence of prolonged ventilator support than the other approaches [3.75% vs. 9.17 and 12.9%, respectively (*p* = 0.0034)]. Multivariable analysis showed that MT was significantly less likely to have prolonged ventilator time when compared to both MS and CS [OR 0.16; 95% CI 0.04–0.57; and OR 0.48; 95% CI 0.12–1.96, *p* = 0.3, respectively]. There was no difference in the incidence of prolonged ventilator time when MS was compared to CS [OR 1.47; CI 0.65–3.35, *p* = 0.36].

The MT group demonstrated a numerically lower incidence of stroke, reoperation for bleeding, renal failure, and atrial fibrillation, when compared to the MS and CS groups, but these results were not statistically significant (Table 2). There were no deep sternal infections in our patient cohort.

Overall, minimally invasive techniques demonstrated decreased 30-day mortality when compared to conventional sternotomy [mini-thoracotomy 1.5% mortality, mini-sternotomy 1.67%, and conventional sternotomy 5.17% (*p* = 0.13)]. In bivariate analysis, mini-thoracotomy patients tended towards less mortality than conventional sternotomy patients [OR 0.38; 95% CI 0.08–1.01, *p* = 0.073], but multivariable analysis showed no significant predictors for mortality.

## Discussion

Aortic valve operations have become amenable to minimally invasive surgical techniques, as the study of these techniques in comparison to conventional sternotomy has become more apparent in the adult cardiac literature. As results continued to improve, minimally invasive aortic valve surgery became the first-line surgical strategy to treat aortic valve disease at our institution. In addition, our patients now prefer minimally invasive surgical procedures that offer equivalent efficacy, durability, and safety, with faster postoperative recovery. However, many studies still describe increased operative times for minimally invasive techniques, and some are skeptical of the efficacy and safety of these techniques.

Most studies comparing aortic valve replacement techniques have found that minimally invasive operations can provide adequate operative exposure and excellent results in all patient groups, in addition to lower hospital costs [4, 13, 14]. While some studies have reported equivalent outcomes with minimally invasive AVR [3, 15–18], most others have reported significant outcome improvements with minimally invasive techniques, especially for mini-thoracotomy, including lower stoke rates, shorter lengths of stay, shorter ventilator time, less mortality, and lower incidence of renal failure [1, 3, 4, 9, 10, 13, 19–22]. It is important to note that while several studies have investigated this topic, few randomized prospective trials have investigated the risks and benefits of minimally invasive aortic valve surgery. One trial demonstrated equivalent safety with mini-sternotomy and conventional AVR, but further trials are necessary to demonstrate improved outcomes with mini-thoracotomy AVR [23].

Two recent propensity-matched analyses demonstrated significantly decreased complication rates for mini-thoracotomy as compared to conventional sternotomy AVR [6, 24]. These include decreased ICU stay, hospital stay, postoperative ventilator time, postoperative atrial fibrillation incidence, infection rates, and transfusion requirements [6, 24]. The results from these propensity-matched studies suggest that the difference in surgical approach alone is likely to account for the improvement in outcomes.

Our results in this study are consistent with findings in other studies. We demonstrate significantly decreased ICU and overall hospital length of stay with mini-thoracotomy, when compared to conventional sternotomy and mini-sternotomy, as well as significantly decreased postoperative length of stay when compared to conventional sternotomy. This suggests that mini-thoracotomy is the best approach to decrease lengths of stay, presumably by allowing patients to recover faster due to less invasive surgical technique. Length of stay in this study is an aggregate number for preoperative and postoperative length of stay. Many patients are admitted with congestive heart failure preoperatively, which skews the data and makes the lengths of stay appear longer in this study. As our minimally invasive case volume has increased, we have implemented new fast track protocols for aggressive early ICU and hospital discharge, which will be addressed in a future manuscript.

Mini-sternotomy and conventional sternotomy patients in our study were significantly more likely than mini-thoracotomy patients to experience prolonged ventilator time. Although nonsignificant, the mortality rate and the incidence of other postoperative complications, like stroke, renal failure, postoperative atrial fibrillation, and reoperation for bleeding, were lower in the mini-thoracotomy patients when compared to the other two approaches. This, in addition to the significantly decreased ICU, postoperative, and overall lengths of stay, signifies a trend towards improved outcomes and faster recovery with mini-thoracotomy AVR at our institution.

Prior studies have demonstrated increased cardiopulmonary bypass and aortic cross clamp times in minimally invasive approaches as opposed to conventional sternotomy, while some have shown comparable times [2, 3, 6, 12, 25]. In one center’s analysis of their 13-year experience with mini-thoracotomy AVR, they demonstrated decreasing bypass and cross clamp times, ventilator times, and postoperative LOS over time, suggesting that increased experience with mini-thoracotomy AVR leads to improved perioperative outcomes [14, 26]. Thus, to achieve better outcomes with minimally invasive AVR, centers must be experienced with the operative technique.

In our study, bypass time and aortic cross clamp time for the mini-thoracotomy approach were significantly shorter than those for both mini-sternotomy and conventional sternotomy. This is likely due to the high volume of mini-thoracotomy AVRs that we perform at our center, and thus the experience and expertise of the surgeons. Not all of the surgeons in the group perform mini-thoracotomy AVR, but all are experienced surgeons. The selection of patients to undergo a mini-thoracotomy AVR is biased due to the surgeons’ comfort level. However, the bypass and aortic cross clamp times for mini-sternotomy were significantly longer than that for conventional sternotomy, likely because we perform fewer mini-sternotomy than mini-thoracotomy procedures at our center. Although mini-thoracotomy procedures provide even less exposure, the increased volume of mini-thoracotomy operations allows the surgeons to acclimate to the restricted space and shorten bypass and cross clamp times. The small number of patients converted from mini-thoracotomy or mini-sternotomy to conventional sternotomy were converted to increase exposure and accessibility. We suggest that mini-thoracotomy may be the superior approach for surgeons who have experience with the technique and have mastered the learning curve. The learning curve is fairly steep initially, but patients much prefer the mini-thoracotomy technique and tend to do better in our institution’s experience.

Preoperatively, the mini-thoracotomy patients in this study had significantly lower STS scores than the conventional sternotomy group, but there was no difference when compared to the mini-sternotomy group. Minimally invasive surgery is more often performed in patients with lower risk profiles due to ease of operation and lower complication risk [22]. In some patients with higher STS scores, conventional sternotomy provides the best outcomes by improving visibility and accessibility. However, in patients with left ventricular dysfunction, similar results between minimally invasive and conventional AVR have been shown [27]. At our institution, we have been increasingly providing successful minimally invasive surgeries to patients with higher preoperative risk profiles. The lower STS scores in the mini-thoracotomy group could be interpreted as an expected lower incidence of postoperative complications and mortality. However, lower STS scores should not impact the cardiopulmonary bypass and cross-clamp times for isolated AVR, both of which were lower for mini-thoracotomy patients. In addition, the mean STS scores for all three groups were within the low-risk range (all were 3.0% or less), indicating that the significant difference may not be clinically relevant. In the future, propensity-matching may be helpful to match STS scores between mini-thoracotomy and full sternotomy patients and compare outcomes if the study has larger sample sizes. In addition, we have begun performing mini-thoracotomy AVR in higher-risk patients, so it would be useful to compare results after mini-thoracotomy in high and low-risk patients. In addition, it would be useful to compare outcomes after mini-thoracotomy and full sternotomy in high-risk patients alone, and include long-term results in future studies.

Lastly, the number of bioprosthetic valves implanted during our study time period far outweighs the number of mechanical valves implanted. In our demographic, most patients prefer a bioprosthetic valve that would obviate the need for Coumadin in most instances, even if they require a reoperation in the future, or a valve-in-valve transcatheter AVR.

In our study, the improved operative times for mini-thoracotomy AVR can likely be attributed to the approach. As more conclusive trials are conducted, the minimally invasive approach, and especially the mini-thoracotomy approach, will likely be substantiated as a technique that minimizes surgical trauma, reduces recovery times, and improves outcomes.

This study reflects similar limitations to other studies in that it represents a single-center experience and was conducted in a retrospective fashion. Further limitations of this study include the short follow-up time (30 day follow-up). These limitations speak to the need for a large prospective study to compare minimally invasive techniques, especially mini-thoracotomy, to conventional techniques.

## Conclusions

Minimally invasive aortic valve replacements, including mini-thoracotomy and mini-sternotomy approaches, are a safe and effective alternative to conventional sternotomy, demonstrating no significant difference in postoperative complications. The mini-thoracotomy approach results in significantly improved operative and recovery times compared to mini-sternotomy or conventional sternotomy.

## Abbreviations

AVR: Aortic valve replacement
CPB: Cardiopulmonary bypass
CS: Conventional Sternotomy
ICU: Intensive care unit
IQR: Interquartile range
MS: Mini-sternotomy
MT: Mini-thoracotomy
STS: Society of Thoracic Surgeons
TEE: Transesophageal echocardiography
